# MSCs loaded with oncolytic reovirus: migration and in vivo virus delivery potential for evaluating anti-cancer effect in tumor-bearing C57BL/6 mice

**DOI:** 10.1186/s12935-021-01848-5

**Published:** 2021-05-01

**Authors:** Seyed-Mahmood Seyed-Khorrami, Hoorieh Soleimanjahi, Sara Soudi, Ala Habibian

**Affiliations:** 1 Department of Virology, Faculty of Medical Sciences, Tarbiat Modares University, Tehran, Iran; 2 Department of Immunology, Faculty of Medical Sciences, Tarbiat Modares University, Tehran, Iran

**Keywords:** Drug Delivery Systems, Targeted therapy, IFN-γ, Anti-tumor effect, MOI, Virus titer

## Abstract

**Background and aims:**

Several oncolytic viruses applications have been approved in the clinic or in different phases of clinical trials. However, these methods have some rudimentary problems. Therefore, to enhance the delivery and quality of treatment, considering the advantage of cell carrier-based methods such as Mesenchymal Stem Cells (MSC) have been proposed. This study was designed to evaluate the performance and quality of cancer treatment based on MSCs loaded by oncolytic reovirus in the cancerous C57BL/6 mouse model. Also, we evaluated MSCs migration potency in vitro and in vivo following the oncolytic reovirus infection.

**Methods:**

C57BL/6 mice were inoculated with TC-1 cell lines and tumors were established in the right flank. Mice were systemically treated with reovirus, MSCs-loaded with reovirus, MSCs, and PBS as a control in separated groups. Effects of infected AD-MSCs with reovirus on tumor growth and penetration in the tumor site were monitored. All groups of mice were monitored for two months in order to therapeutic and anticancer potential. After treatments, tumor size alteration and apoptosis rate, as well as cytokine release pattern was assessed.

**Results:**

The results of the current study indicated that the effect of reovirus infection on AD-MSCs is not devastating the migration capacity especially in MOI 1 and 5 while intact cells remain. On the other hand, MSCs play an efficient role as a carrier to deliver oncolytic virus into the tumor site in comparison with systemic administration of reovirus alone. Apoptosis intensity relies on viral titration and passing time. Followed by systemic administration, treatment with oncolytic reovirus-infected AD-MSCs and MSCs alone had shown significant inhibition in tumor growth. Also, treatment by reovirus causes an increase in IFN-γ secretion.

**Conclusion:**

The results of in vitro and in vivo study confirmed the tumor-homing properties of infected AD-MSCs and the significant antitumor activity of this platform. Hence, our results showed that the cell carrier strategy using oncolytic reovirus-loaded AD-MSCs enhanced virus delivery, infiltration, and antitumor activity can be effectively applied in most cancers.

## Background

The accumulation of genetic alterations may lead to cancer development. Various cancer types were reported as the second cause of deaths worldwide, leading to 9.6 million global mortality [1, 2]. Despite the promising advances of existing methods in cancer therapy, these strategies confront some challenges such as low efficiency as well as side effects [3, 4].

Nowadays, besides the conventional methods, several types of research have been launched to develop novel therapeutic approaches to fighting more efficiently against cancer. For instance, the natural oncolytic properties of some viruses or genetically modified viruses against various tumors have been considered as a potential treatment for cancer [5–7].

The most significant oncolytic potency has been reported mainly in adenovirus, reovirus, vesicular stomatitis virus, measles virus, Newcastle disease virus, and HSV-1 [5, 6, 8].

The first report which revealed that wild-type reovirus has a brilliant potential oncolytic property only within mammalian transformed cells were documented in 1977 [9, 10]. Sensitivity to reovirus lysis has occurred when double-stranded RNA-activated protein kinase (PKR) is deactivated because of the activated Ras signaling pathway in several tumor cells [9, 11, 12]. Considerably, in several undergoing clinical trial phases, reoviruses are used as an oncolytic virus (OV) in the treatment of various human tumors [7, 12–15].

When reovirus was systemically administered in the patient’s body, OVs efficiently could target metastatic cancer cells. The major complication for usage of OVs is delivery issues. The efficacy of systemic administration of OVs is often diminished by circulating neutralizing antibodies and immune cells. Disadvantages and advantages of anti-cancer therapeutic application of reovirus are listed in Table 1 [6, 16]. The utilization of cellular vehicles is proposed to encounter this issue and reduce the further probable adverse effects [17, 18].

**Table 1:** Advantages and disadvantages of oncolytic reovirus (T3D) for cancer therapy

	Role Mechanism of action	Advantages	Disadvantages
oncolytic reovirus (T3D)	Oncolytic agent/ Direct lysis Immune cell recruit Immune cells priming	Systemically/locally administration [40] Targeting metastatic cancer cells [41] Poor adverse effects Well tolerable dosage Mild and asymptomatic [42] Specifically replicate in cancer cells with an activated Ras pathway and affinity to RAS mutant cancer cells but not in normal tissue [41–43] Selectively replicate in cancer cells [44] Selectively cytopathic to many human cancer cells [42] Priming of antitumor immunity Launch an immune response against cancer cells [45, 46] Induce apoptosis via triggering intrinsic/extrinsic pathway [42]	Immune-mediated neutralization both antibodies and immune cells [47, 48] Off-target effect [47] Delivery issues [40]

Mesenchymal stem cells (MSCs) are multipotent fibroblast-like cells that can be isolated from several different tissues [19–21]. In several studies, MSCs have been recognized as a capable carrier for the delivery of anti-cancer agents for their strong inherent tropism into the tumor microenvironment. They can regulate the tumor environment and can interact with tumor cells in numerous pathways by suppression of local immune response, inhibit tumor growth, via inhibition of angiogenesis, induction of cell cycle arrest and apoptosis as well as the support of viral replication. These important features of MSCs make them attractive candidates for OVs delivery. MSCs also shield the pre-loaded virus from immune-mediated neutralization during their re-localization to tumor sites [22].

The several studies show that MSC-mediated delivery of OVs is a reassuring approach for achieving synergistic anti-tumor efficiency with recuperation safety profiles. A summary of the advantages and disadvantages of MSCs applications as a cell carrier was represented in Table 2.

**Table 2:** Advantages and disadvantages of mesenchymal stem cells for cell-based therapy

	Role Mechanism of action	Advantages	Disadvantages
Mesenchymal Stem Cell	Cell-Carrier Delivery Immunomodulatory	Availability; easy to isolate, growth and expand in culture [19–21, 25] Systematically/ locally administration [26] Resolve delivery issues of OV into tumoral and possible metastatic site: (protect the pre-loaded virus from immune-mediated neutralization and support viral replication) [19, 27–31] Natural capability to mobilize, migrate and homing [3, 32, 33] Inherent anti-cancer effects (suppress tumor growth) [27] Affecting the microenvironment of tumors [21, 33–35] Limited replicative lifespan (guarantees safety from the threat of malignant formation) [27] Inhibition of angiogenesis in tumoral zone [27] Deep infiltration in tumor ECM and overexpress CXC chemokine receptor 4, 7 (CXCR4, CXCR7) in response to a different set of chemokines in tumor area [8, 36–38]	Differentiation inside of the body [39] Tumor promoting abilities in some cases [27] Accumulation in the lung [26]

According to registered data on the US National Institutes of Health clinical trial database (http://www.clinicaltrial.gov/), there are 949 MSC-based clinical trials studies up to the late 2020, either have been accomplished or still ongoing all over the world [23, 24].

Also, reducing the main concern about the possible side effects of MSCs-therapy by using infected-MSCs is of paramount importance. In this regard, to appliance MSC as an oncolytic reovirus carrier, it must be examined to be sure that it can keep its natural properties toward tumors and support anti-tumor activity.

The increasing prevalence of cervical cancer and its high mortality rate have forced scientists to find more effective therapeutic approaches. Based on MSCs properties and the anti-cancer impacts of Reovirus, the aim of this study was to examine the migration capacity of infected MSCs with oncolytic reovirus in vitro and the evaluation of the anti-tumor effects of oncolytic reovirus-loaded MSC in cervical cancer model via numerous methods including apoptosis and immune response measurement, that could open up new windows to combined therapy to achieve the excellent outcome in fighting to cancers.

## Materials and methods

### Isolation of AD-MSC from mice

Adipose-derived MSCs (AD-MSC) were isolated from C57BL/6 mice according to a previously published protocol (124). Healthy female C57BL/6 mice (6- to 8-weeks old) were purchased from Pasteur Institute, Tehran. The animals were kept and handled under the standard laboratory conditions in terms of the guidelines of the ethical committee of Tarbiat Modares University. This study was approved by the Iran national committee for ethics in biomedical research with the code number of IR.TMU.REC.1395.546.

Briefly, mice were sacrificed and abdominal adipose tissue was isolated under sterile situations. The adipose tissue is well minced and then digested with type I collagenase (1 mg/ml; Gibco, USA, Lot: 1915460) at 37 °C for 10–15 min. Undigested tissue was removed and the supernatant centrifuged for 5 min in 1500 rpm at room temperature. The cell pellets were resuspended in high-glucose Dulbecco’s Modified Eagle’s Medium with F12 (DMEM-F12-Atocel, Austria) which supplemented with 15% fetal bovine serum (FBS; Gibco, Carlsbad, CA) and 1% penicillin/streptomycin (P/S). The cells were plated in a T75 flask (SPL) and incubated at 37 °C and 5% CO_2_. After 24 h non-adherent cells were removed. The majority of adherent cells are the homogenous population of MSCs in morphology and expression of cell surface markers through 3 passages and used for all following experiments.

### Characterization of AD-MSCs

To determine the phenotypic characteristics of the AD-MSCs, the expression of CD45, CD34, CD29, CD73, and CD90 cell surface markers were examined by flow cytometry using antibodies purchased from eBioscience (Table 4).

Furthermore, isolated AD-MSCs were cultured in adipogenic and osteogenic induction medium for 21 days and their differentiation potential to adipocyte and osteocyte was determined by Oil Red O (Sigma Aldrich) and alizarin Red S (Sigma–Aldrich) staining, respectively.

### Oncolytic reovirus propagation, titration, and AD-MSCs infection protocol

The oncolytic reovirus that is isolated from wild-type reovirus type 3, Dearing strain (T3D), was provided by the department of medical virology of Iran University. Reovirus stock was propagated and tittered by the standard protocol of tissue culture infectious dose 50 (TCID50) endpoint assay on L929 cells that were obtained from the Pasteur Institute of Iran.

To calculate the reovirus titer, viral serial dilutions were prepared from the virus main stock. The confluent L929 cells in cell culture plate 96 wells are infected with the prepared viral serial dilutions and after two hours of incubation and gentle shaking of the plate to attach the virus to the cells, the supernatant is removed and replaced with fresh DMEM medium containing 2% serum. After 72 h, the cytopathic effect is observed and recorded in each well, and then the virus titer is calculated using the method of Reed & Muench. It should be noted that the cytopathic effect results were considered by comparing with negative control (non-infected) and positive control (inoculated with an undiluted virus).

For reovirus inoculation, AD-MSCs cells were seeded into 6-well plates at a density of 3 × 10^5^ cells per well. The confluent cells were infected with different MOI of the virus in serum-free medium. After 2 h incubation at 37 °C with shaking for adsorption of the virus, the inoculum was replaced with the appropriate fresh DMEM with 2% FBS culture medium.

### The effect of infected AD-MSCs by reovirus

MSCs were grown under the standard situation in 6-well plates and treated by reovirus in the different multiplicity of infection (MOI) [1, 5, 10]. The treated MSCs were subjected to apoptosis assay at 24, 48, and 72 h post infection (P.I) using Annexin V-7AAD apoptosis detection kit according to the manufacturer’s instructions (eBioscience, USA, Lot:4,331,154).

The medium is removed from 6 well plates by aspiration and the MSCs were washed with PBS.

The cells were detached through trypsin/EDTA treatment. Approximately 2 × 10^5^ cells were rinsed with PBS, and then with binding buffer and stained with Annexin V-PE (eBioscience, MA, USA) and 7-AAD (eBioscience, San Diego, CA, USA) according to the manufacturer’s protocols. Flow cytometric analysis was performed (BD Biosciences, Mississauga, ON, Canada), while data were analyzed using FlowJo™ software (Tree Star Inc, USA). To avoid confounder fluorescence from dead cells, live cells were gated tightly and compensation was done.

### Effect of reovirus infected AD-MSCs on autophagy and apoptosis-associated gene expression

Furthermore, the content of another plate that infected MSCs via different MOI of reovirus was detached through trypsinization and the rate of apoptosis and autophagy was assessed by a StepOnePlus™ real time PCR system (Applied Biosystems) (Biofact, 2X high ROX, Cat. No. DQ385-40 h) according to the manufacturer`s instruction (Total RNA prep kit, BioFact Cat. No. RP101-100) for three genes including BAX, LC3 and P53 and HPRT1 as the housekeeping reference gene for normalizing. The expression level of each target gene was determined by the 2^−ΔΔCT^ method. Primers list included in Table 3.

**Table 3:** Sequences of the forward and reverse primers

Gene, Premier		Sequence
P53	Forward	CCGAAGACTGGATGACTGC
	Reverse	GTCTCGGTGACAGGGTCC
BAX	Forward	CAGCGGCAGTGATGGAC
	Reverse	TCCTGGATGAAACCCTGTAG
Bcl-xL	Forward	CAGTCAGCCAGAACCTTATC
	Reverse	AACACCTGCTCACTTACTGG
HPRT1	Forward	TCCCAGCGTCGTGATTAG
	Reverse	CGAGCAAGTCTTTCAGTCC
Reovirus (L3 gene)	Forward	CGCGTCCTCAATTTTGGGTAAAC
	Reverse	CCGCCGTCTTTTGGATATGAACTA
Beclin 1	Forward	ACAGCCCAGGCGAAACCAG
	Reverse	CCTCCCCGATCAGAGTGAAGC
mLC3	Forward	CGCCGACCGCTGTAAGGAG
	Reverse	GCGCCGGATGATCTTGACC

### Analysis of the effect of reovirus on the migration potential of AD-MSCs

Migration potential of AD-MSCs was measured by both scratch assay and CXCR4 expression. For the scratch assay, MSCs were cultured at 6-well plates and infected at MOI: 0, 1, 5, and 10 with oncolytic reovirus. Then, the MSC monolayer manually was scratched with a 100 µL sterile micropipette tip that formed a void space. Twenty-four hours later, the wound recovery in the plates was visualized under inverted microscopy and the image of the scratched area of experimental groups was captured for further analysis. Images were captured in triplicate for precise analysis. The macroscopic size of wound healing was calculated using ImageJ software. Data were calculated as mean ± SD.

In addition, the CXCR4 expression as an indicative marker of migration was measured in reovirus infected MSCs using CD184 (CXCR4) Monoclonal Antibody (2B11), APC, eBioscience™ (Cat. No. 17–9991-82) by flow cytometry method.

### Establishment of a subcutaneous cancer model and administration studies

The TC-1 cell line (ATCC^®^ CRL­2785™) was provided by Pasteur Institute of Iran and was harvested to reach 80% confluence. A total of 1 × 10^6^ TC-1 cells were subcutaneously injected into the right flank of mice. Following 7–9 days, tumors were palpable. Mice were randomly distributed into five experimental groups consisting of 10 animals per each group. Subsequently, the flank tumor was treated by intravenous injection of three serial doses of 5 × 10^5^ MSCs (Msc group), 5 × 10^5^ reovirus-loaded MSCs (Msc-Reo group), reovirus (Reo group) (equal virus particle to MOI 5), PBS (Pbs control group) and, healthy control mice were considered as a control group by inoculating 5 × 10^5^ reovirus-loaded MSCs (Msc-Reo-nT group) at 72 h intervals. All animals were weighed separately and tumor dimensions were also measured every four days following treatment to calculate tumor volumes by the formula: volume = (W^2^ × L)/2.

In order to investigate the therapeutic process, three mice were randomly selected from each group before the next injection. These mice were sacrificed and subsequently exposed to dissection. Their spleens and the visible tumor nodules were removed. Tumors were processed to detect elevated rates of apoptosis.

Furthermore, mouse survival was monitored daily during the experimental period, and the animal should be culled under tumor growth circumstances if tumor size exceeds 2000 mm3 and impedes animal mobility.

### Mouse splenocytes isolation

After removing the mouse spleen, it was placed within a petri dish along with 2 mL medium (RPMI 1640 + 5% FBS) and teased the spleen with the bottom of the syringe plunger until dissolve all cells and no more than stromal tissues remain. The spleen cells were rinsed twice with PBS under centrifugation at 2000 rpm, 10 min. Aspirate supernatant completely in every stage and finally removed red blood cells by lysis buffer. Then spleen cells were washed again with PBS and centrifuged the same as previously mentioned. The supernatant was discarded and the pellet resuspended in 1 mL RPMI 1640 and subjected to the accurate assessment of cell count.

### LDH cytotoxicity assay

To determine and analyze cell death lactate dehydrogenase (LDH)-based cytotoxic T lymphocyte (CTL) assays were performed. TC-1 cells and T lymphocyte cells of the spleen cells were plated (5 × 10^4^ and 125 × 10^4^ cells per well, respectively) simultaneously in 96-well plates. Only allocated wells to the background are seeded without TC-1 cells. The plate was incubated for 4 h at 37 °C and 5% CO_2_. Other steps were performed according to the manufacturer’s instruction of LDH kit (BioLegend, Cat. No: 426401). Finally, all wells absorbance was measured at 490 nm.

### Cytokine assay

The enzyme-linked immunosorbent assay (ELISA) is the most approved method to detect cytokines. Splenocytes (5 × 10^6^ cells per well) were seeded in 12-well plates to assess cytokines due to encounter with PBS as mock, specific, and non-specific antigens, HPV-E7, and phytohemagglutinin (PHA), respectively. Seventy-two-hour post-incubation and stimulation, all specimens' supernatants were collected separately to measure IL-6, IL-10, TGF-β, and IFN-γ in accordance with an approved protocol for ELISA assay. Briefly, the mentioned antibodies were coated at 96-well plates. After twice washing, BSA was added and incubated for one hour at room temperature. Three times washing was performed, and all specimens were inoculated into wells and incubated again at room temperature. The secondary antibodies were added and incubated for 90 min followed by three times washing. Subsequently, the horseradish peroxidase (HRP) solution was placed in all wells and incubated in dark for 45 min. HRP was removed and its substrate, tetramethylbenzidine (TMB), was added after washing three times. Finally, by adding TMB to each well, the blue-colored products were generated and followed by reading the absorbance at 450 nm (The cytokines listed in Table 4).

**Table 4:** Specifications of all antibodies used in AD-MSCs immunophenotyping and cytokine assays

Antibody	Catalogue no	Company	Dilutions titer
Mouse IFN-gamma DuoSet ELISA	DY485-05	R & D systems	Capture 4 µg/ml Detection 400 ng/ml
Mouse IL-10 DuoSet ELISA	DY417-05	R & D systems	Capture 4 µg/ml Detection 300 ng/ml
Mouse TGF-β DuoSet ELISA	DY402-05	R & D systems	Capture 4 µg/ml Detection 75 ng/ml
Mouse IL-6 DuoSet ELISA	DY406-05	R & D systems	Capture 2 µg/ml Detection 50 ng/ml
Anti- mouse CD34-FITC	11-0341-82	e-Bioscience	1 µg/test
Anti- mouse CD45- FITC	11-0451-82	e-Bioscience	0.5 µg/test
Anti- mouse CD29-PE	12-0291-82	e-Bioscience	1 µg/test
Anti- mouse CD73 -PE	12-0731-82	e-Bioscience	0.125 µg/test
Anti- mouse CD90-PE	12-0902-82	e-Bioscience	0.5 µg/test
Rat IgG2a kappa Isotype Control- FITC	11-4321-42	e-Bioscience	1 µg/test
Rat IgG2b kappa Isotype Control- FITC	11-4031-85	e-Bioscience	1 µg/test
Rat IgG1 Isotype Control—PE	12-4301-82	e-Bioscience	0.125 µg/test
Rat IgG2a kappa Isotype Control -PE	12-4321-80	e-Bioscience	0.5 µg/test

### In vivo MSC migration assay

As known, MSC can migrate toward the inflammatory site of the body. Thus, a method to properly evaluate MSC migration through Carboxyfluorescein succinimidyl ester-labelled AD-MSC was performed, and followed by trypsinization of MSCs flask, all cells were labelled by CFSE, according to the labelling protocol [49]. After proper incubation time, CFSE-labelled AD-MSCs were ready to inject into mice through the tail vein. About thirty hours later, the injected mouse was sacrificed and dissected to isolate internal organs including lung, spleen, lymph nodes in peripheral of tumor, intestine and tumor to indicate MSCs migration by flow cytometry.

### In vivo apoptotic gene expression

The sections of tumors were prepared and minced adequately to digest with the enzyme. This suspension was centrifuged, the supernatant was discarded and the pellet was rinsed with PBS twice. Half of this cell suspension were labelled using Annexin V-7AAD apoptosis detection kit according to the manufacturer’s instructions (eBioscience, USA) to determine apoptotic stages by flow cytometry.

### Tracking viral genome in tumor sites

To verify the MSCs could be used as an effective carrier for reovirus delivery to tumor sites, the tracing of the reovirus genome in tumor tissue is evaluated. Tumors in each group smashed and the viral genome was extracted by a viral extraction kit (GeneAll, Korea, Cat. No. 128–150) and then PCR was performed according to previously published data. The primers used for amplification of reovirus major capsid protein lambda 1 (L3 gene segment) (Table 3) [50].

### Statistic analyzing

All values are expressed as the mean ± SD (standard deviation). Statistically significant were assessed by Student's t-test between two groups, ANOVA, and post-hoc Tukey's test for comparing results in different groups. All analyses were performed using R version 4.0.1 and GraphPad Prism v.8.0. A p-value < 0.05 was considered statistically significant. Experiments were performed in triplicate and repeated three times with similar results.

## Results

### Immunophenotypic profile of AD-MSCs showed differentiation of cells into adipocyte and osteocyte

**Fig. 1:**
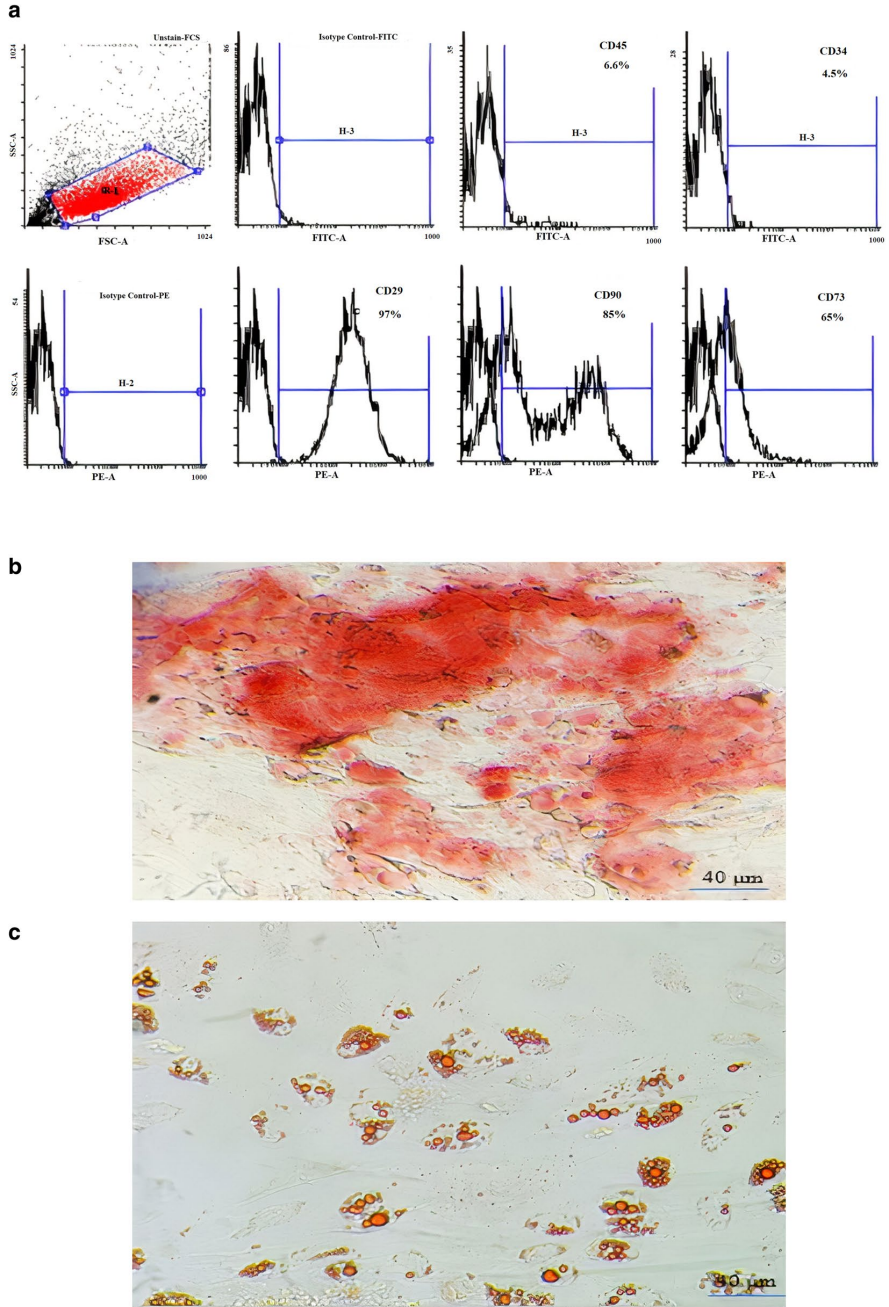
Characterization, immunophenotyping and differentiation potential. **a** Immunophenotyping of AD-MSCs by flow cytometry. The CD Markers expressed by AD-MSCs cells in high levels including CD90, CD29, and CD73 versus low expressed hematopoietic markers such as CD45 and CD34. **b** The microscopy image of differentiated osteocytes deposited calcium indicated in red color. **c** The microscopy image of differentiated adipocytes including lipid droplets in red color

After each AD-MSCs isolation, the immunophenotype of isolated cells at passage 3, was examined by flow cytometry. Flow cytometry results were analyzed using flowing software. An accurate gate was created around all detected cells that had the common characteristic of MSCs according to FSC-SSC dot plots. Then, the expression of different CD markers in the gate population was determined using overlay histograms, indicated isotype control, and anti-CD marker staining result. The percentage of the positive population relative to the control isotype was reported as the expression level of each CD marker. An example of the flow cytometry result of AD-MSCs immunophenotyping is shown in Fig. 1a. Finally, the mean ± SD of each CD marker expression level was calculated. According to the obtained results, AD-MSCs expressed 2.6 ± 2% CD45, 2.3 ± 1.5% CD34, 98 ± 2% CD29, 80 ± 6% CD90 and 65 ± 2% CD73 cell surface markers.

Moreover, the differentiation potential of AD-MSCs post 21 days culture in the osteogenic and adipogenic induction media were assessed using alizarin red and oil red O staining, respectively. Figure 1b, demonstrated the homogenous differentiation of AD-MSCs into adipocytes including lipid droplets. Figure 1c confirmed the differentiation potential of AD-MSCs into osteocytes indicated by calcium deposition.

### Reovirus increases apoptotic cells in MSCs

**Fig. 2:**
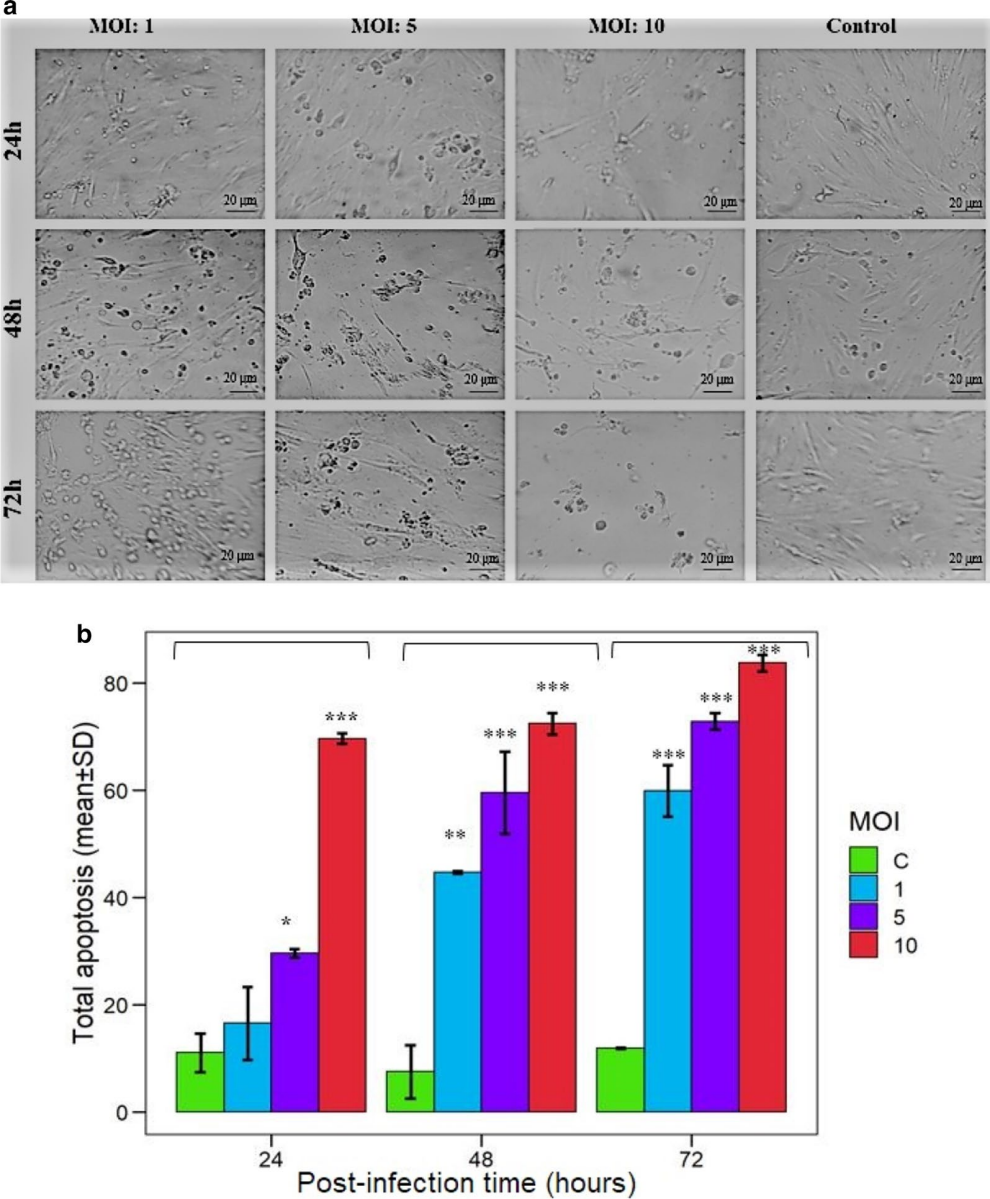
Evaluation of MSCs apoptosis by different MOI of reovirus. **a** Representative microscopic images of reovirus cytopathic effect on MSCs in both: different MOIs (Control, 1, 5 and 10), and incubation time (24, 48, and 72 h P.I). **b** The mean percent of AD-MSCs apoptosis were represented at 24-, 48-, and 72-h post-infection with reovirus at different MOIs (Control, 1, 5, and 10). In this study, induced total apoptosis rate was significantly different among the control group (PBS) and reovirus-infected MSCs groups in terms of MOIs (1, 5, and 10) and hours.P.I (24, 48, and 72 h P.I) (P-value < 0.05). Significant differences in total apoptosis rates among the three mentioned MOIs at 24,48, and 72 h P.I were observed, but the difference between 48 and 72-h P.I was observed only in MOI 10 (P-value: 0.0111). This experiment was repeated three times and the results were reported as mean. According to Tukey's post hoc test, significant differences (P-value < 0.05) between groups are determined (*: P ≤ 0.05; **: P ≤ 0.01; ***: P ≤ 0.001)

In order to determine the effect of the reovirus infection on the apoptosis of mesenchymal stem cells, the AD-MSCs were infected with the virus at MOI 0, 1, 5, and 10 and were evaluated at intervals of 24, 48, and 72 h after infection. As the microscopic examination of different test groups shows, the presence of the reovirus causes cell destruction and death, which is directly related to the titer of the virus and the time of exposure (Fig. 2a). Assessment of annexin V expression on AD-MSCs by flow cytometry confirmed the induction of apoptosis by the reovirus. As shown in Fig. 2b, 24 h after infection, 18, 30, and 71% apoptosis are induced in the MOI of 1, 5, and 10, respectively. The rate of apoptosis in each group increases significantly (P < 0.05) at 48 h and 72 h compared with 24 h post-infection, depending on the virus shedding into the culture medium. According to statistical analysis, there is a significant difference (P < 0.05) in apoptotic induction between different MOIs at the same intervals. The maximum level of apoptosis in AD-MSCs is observed at 72 h after exposure to the reovirus were 60, 74, and 83% in the MOI 1, 5, and 10, respectively.

### Reovirus enhances in vitro expression of apoptotic and autophagy genes and reduces anti-apoptotic genes

In several reports, it had been declared that the reovirus could affect both apoptotic and autophagy pathways. Expression of apoptotic genes (P53 and BAX), and autophagy gene (LC3) were evaluated in both control and infected-MSCs groups in different MOIs (control, 1, 5 and 10) and incubation time (24, 48 and 72 h P.I) by real-time PCR. The expression of BAX apoptotic gene increased after infection of MSCs with reovirus. At 72 h P.I, a significant difference was detected, especially in MOI 10 in comparison with both MOI 1 and 5 according to the significance of P-value: 0.0002 and P-value < 0.0001, respectively. The expression level of LC3 becomes significant within 72 h P.I (P-value: 0.0006), especially in MOI 10 compared to MOI 1. In MOI 5, high levels of LC3 were expressed 72 h P.I (P-value: 0.0029) compared to MOI 1. The rate of P53 expression becomes significant via high MOI and passing time from inoculation in all infected groups, so in 72 h P.I (P-value: 0.00015).

**Fig. 3:**
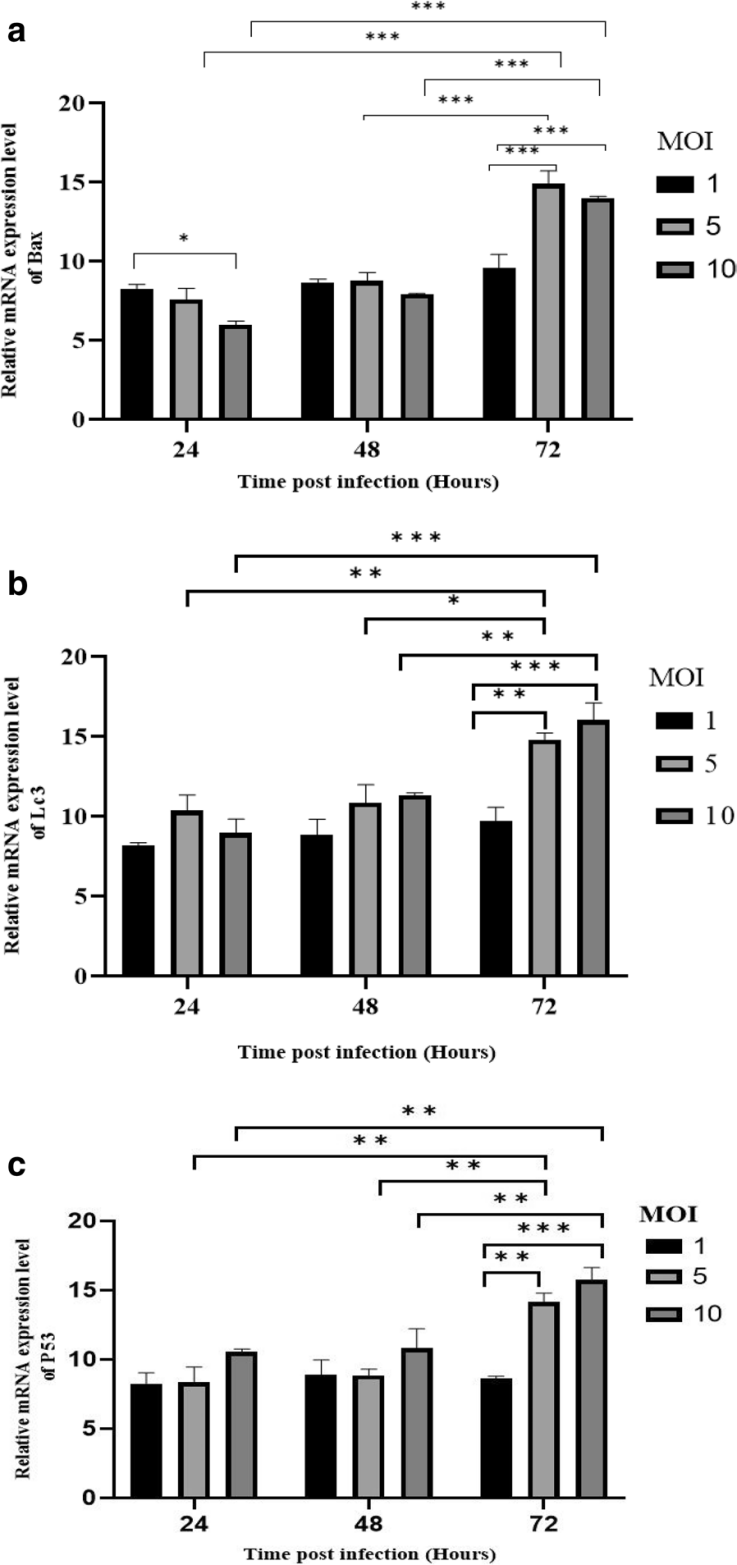
The expression of BAX, LC3, and P53 in infected MSCs with reovirus in different MOIs and incubation time. **a** The expression of BAX increased via reovirus infection. Statistically**,** the significance level is obvious among MOIs in 72 h post-infection time except between MOI 5 and 1 (P-value > 0.05. **b** The LC3 expression in reovirus infected MSCs is increased via high MOIs. The post-infection time is an important factor that influences results (P-value < 0.05). **c** After infection of MSCs with reovirus, the expression of P53 increased. In both 48- and 72-h post-infection significant results among different MOIs were found, (P-value: 0.000). The Experiments were performed in triplicate and graphs were determined by GraphPad v.8.0.2 and analyzed by Tukey's post hoc test. (*: P ≤ 0.05; **: P ≤ 0.01; ***: P ≤ 0.001)

The overexpression of apoptotic genes was begun at 48 h P.I in MOI 10 (P-value < 0.05). We found that the oncolytic reovirus induced both apoptosis and autophagy pathways in high levels in both MOI 5 and 10 after 72 h P.I (Fig. 3).

### Reovirus reduces migration ability of MSCs especially in MOI 10

To evaluate the migration potency of MSCs that are influenced by a viral infection, surface CXCR4 marker was assessed by flow cytometry, and scratch assay was performed in vitro.

**Fig. 4:**
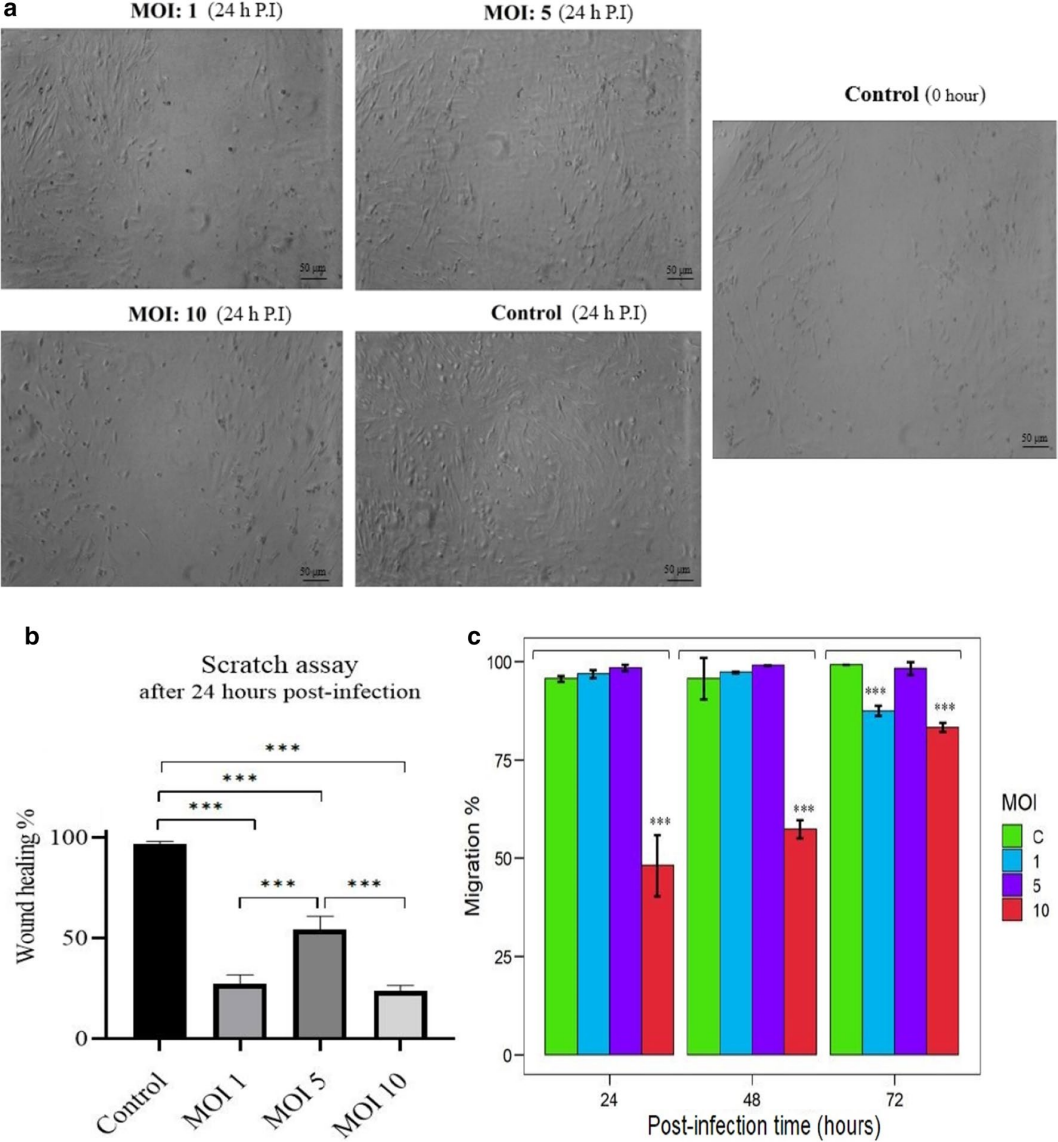
Determination of MSCs migration potential following treatment with oncolytic reovirus in vitro. **a** Representative images of cell migration in the scratch assay of MSCs with or without reovirus infection through different MOI 0 (Control), 1, 5, and 10 in 24 h P.I. There were many migrated cells in the scratched space after 24 h depended on cell integrity. Images were captured in triplicate for precise analysis. **b** After 24 h P.I, the wound healing rates were assessed. Images were captured in triplicate for precise analysis. Macroscopic size of wound healing rates was calculated using ImageJ software. Data were calculated as mean ± SD. In the first 24 h P.I, the relative reduction in wound healing was assessed. Abrupt activation of NF-κB via reovirus infection can trigger the expression of a wide range of genes that are engaged in the cell migration process. The high MOI [10] damage cell integrity that subsequently has an adverse effect on cell mobility. **c** The migration potency of infected MSCs was analyzed by the evaluation of surface CXCR4 marker in variant MOI (control, 1, 5, and 10) and time (24, 48, and 72 h P.I) by flow cytometry. In MOI 10 reovirus showed a destructive effect on MSCs and declined results on migration capacity (P-value: 0.0106). This experiment was repeated three times and statistically, the analysis was reported by Tukey's post hoc test. (***: P ≤ 0.001)

After the reovirus inoculation (different MOI) and scratching monolayers of MSCs wound healing was monitored by an inverted microscope after 24 h post scratching. In the control group (PBS) the scratched area was proximately fully covered by migrated MSCs and in MOI 5 the migrated MSCs were obvious. In MOI 1 and 10, only a few MSCs were observed in the interval space (Fig. 4a).

After 24 h P.I, a significant declined level in CXCR4 expression was shown only in MOI 10 (P-value: 0.000) which is affected by cellular arrest (disruption of cellular protein expression) and severe triggering of apoptosis as a consequence of robust virus invasion. In MOI 10, a similar pattern was repeated in 48 and 72 h P.I. In MOI 5, the amount of CXCR4 marker in infected cells and in healthy cells during indicated times of infection are expressed similarly at a high level. Based on represented data, MOI 5, up to 72 h, P.I have no undesirable influence on reovirus infected-MSCs migration marker (P-value > 0.05) (Fig. 4a).

## In vivo results

### The MOI 5 was chosen as an efficient MOI for sensible migration ability and apoptosis rate up

Series of assessments performed in vitro to find the best exposure MOI and injection time (two main influential factors) for in vivo investigations. In MSCs, apoptosis rate and migration the potency of MSCs was assessed in 24, 48, 72 h P.I by reovirus in different MOI (1, 5, and 10) through flow cytometry. Based on the obtained in vitro experimental results and analyzing of data, we concluded to exclude the MOI 10 of reovirus from the study due to observing the high level of apoptosis in this MOI that rapidly causes the loss of vividness and viability of the MSCs.

The MOI 1 was also excluded from the study due to insignificant viral progeny and the lower rate of apoptosis compared to MOI 5 (the essential factors to eliminate the risk of MSCs persistence in the body).

The MOI 5 was chosen as an efficient MOI due to its adequate and sensible apoptosis rate up to 48 h P.I and keeping MSCs survival up to 72 h P.I, as well as maintaining cell migration status at 48 h equivalent with intact MSCs.

For in vivo experimentations, MSCs became infected with MOI 5, detached, and counted after 48 h P.I and injected into mice bodies via the lateral tail vein route.

This process was programmed for three consecutive days and the normalization of the number of stem cells as well as the injected virus particles, which performed according to the literature and our previous experiments. Mice were sacrificed 3 (that took only the first treatment), 6 (two treatments), and 13 days (all three treatments) after the first injection. Subsequently, several experiments were performed to investigate differences in obtained results among groups.

### The highest level of cytotoxicity rates was shown in MSC related groups

Cytotoxicity was performed on isolated splenocytes from the mice spleen by LDH assay to determine lymphocyte reaction to the specific and non-specific antigens. The released LDH levels assessed three days after the first treatments by sacrificing three mice per group. It revealed significant differences among the groups (P-value < 0.05). The cytotoxicity rate among the control group (Pbs) compares to Reo, Msc and Msc-Reo groups were significant (0.0307, 0.0074, and 0.0037 respectively).

During the second sacrifice time 3 days after the second treatment, the rate of cytotoxicity among the groups showed no significant difference (P-value > 0.05). The analysis of the released LDH levels on the third sacrifice time (one week after the final treatment) indicated a significant difference among groups compare to control (P-value < 0.001) which was due to a significant difference between groups Msc-Reo and Reo (P-value < 0.0001). Additionally, the same difference was observed among groups Msc and Msc-Reo via Msc-Reo-nT (P-value < 0.0001).

**Fig. 5:**
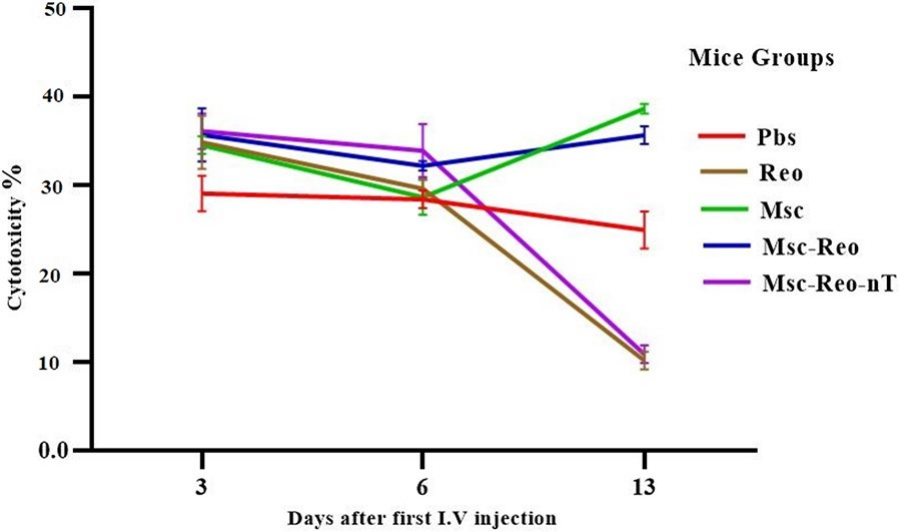
Cytotoxic T lymphocyte response was measured by released LDH. In 3, 6, and 13 days after first treatments, mice were sacrificed then isolated splenocytes were encountered by antigens and cytotoxicity was assessed by LDH assay. First treatments had shown robust cytotoxicity rates in all groups. MSCs could accelerate cytotoxicity in corresponding tumoral groups. This experiment was repeated four times and data were analyzed by Tukey's post hoc test

Analyzes of data showed that only two groups, the Msc-Reo-nT and Reo had a significant difference compare others in released LDH levels gradually over time, both groups showed a declining trend, especially after the final third sacrifice time (Fig. 5).

### High tumor apoptosis rates were shown in MSCs related group (Msc and Msc-Reo groups) by flow cytometry

**Fig. 6:**
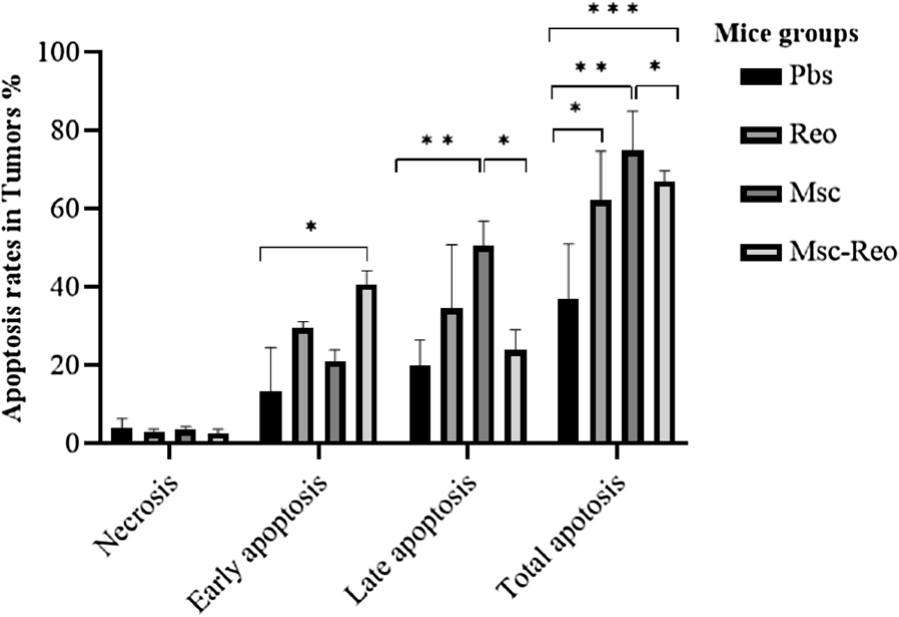
Evaluated apoptosis rate in tumors after treatment in each group by flow cytometry. In both groups containing the reovirus (Reo and Msc-Reo groups), the rate of early apoptosis is higher than the control group, but late and total apoptosis are more significant in the groups containing mesenchymal stem cells. (*: P ≤ 0.05; **: P ≤ 0.01; ***: P ≤ 0.001)

The rate of apoptosis in the tumor was evaluated by flow cytometry. The high amount of total apoptosis was observed in groups Msc, Msc-Reo and, Reo, respectively (Fig. 6).

### Reovirus delays tumor growth and prolongs survival rate in mouse model during treatment

**Fig. 7:**
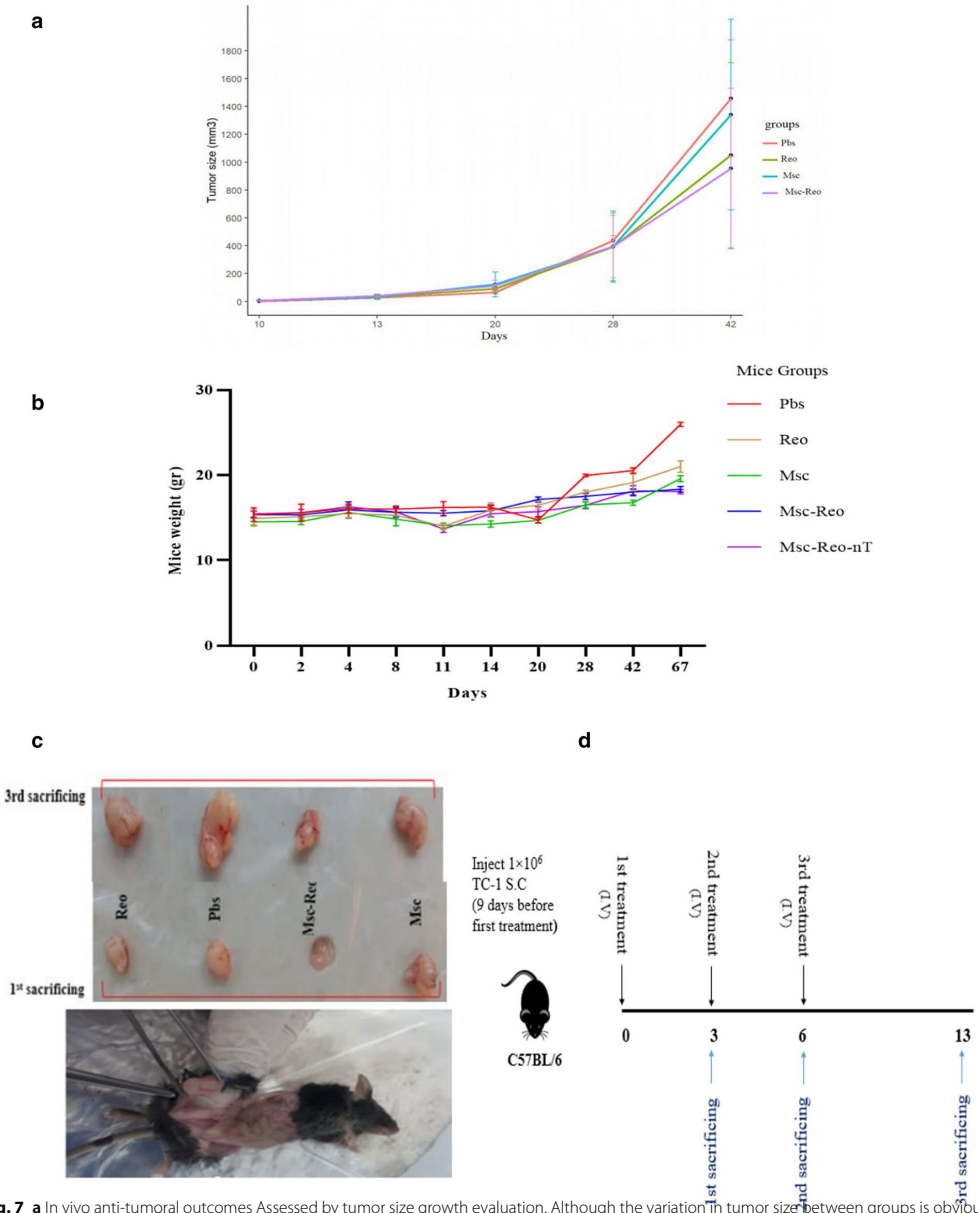
**a** In vivo anti-tumoral outcomes Assessed by tumor size growth evaluation. Although the variation in tumor size between groups is obvious, this variation is not statistically significant. **b** The mice's weight was assessed following treatments. In both Msc-Reo and Msc-Reo-nT groups increasing weight has shown a slow trend compared to other groups. After the final treatment, the control group (Pbs) showed increased weight because tumor growth was raised. The diagram was determined by the mean weight of each group. **c** In vivo anti-tumoral response in the 1^st^ and final sacrifice times depicted by tumor sizes. In 3 (after first treatment) and 13 days (after all treatments) after the first treatments three mice from each group randomly were sacrificed and tumor isolated for more investigation. **d** The time course of the whole project was depicted in **d**. The time of I.V treatments and sacrificed turns indicated by arrows.

Tumor size assessed by the caliper was drawn in Fig. 7a. The ANOVA test was not determined with a significant difference among groups in this element (P-value: 0.510). Interestingly, during treatments tumor size were stationary. Moreover, the mean weights of different groups of studied mice during two months of surveillance informed in Fig. 7b.

It must be considered if tumor size exceeded more than 2000 mm^3^ and prone to bleed and impede in mobility, mice had been culled based on animal ethics protocols.

### A significant increase of cytokines was apparent in MSCs related groups

To evaluate the potency of released cytokines, isolated lymphocytes were stimulated with tumor antigens before and then encountered with mitogen and tumor-specific antigen (E7). Afterwards, the supernatant was collected and finally, ELISA evaluated the levels of released cytokines.

In the first sacrifice time, the amount of IL-6 was significantly different among groups via stimulation with both antigens. All groups produced a higher rate of IL-6 than group Pbs in response to tumor-specific antigen (E7) (P-value: 0.004). In the case of stimulation with specific antigens, only the secretion of group Msc was higher than the control group and was significant (P-value: 0.0175). The significant difference in IL-6 level among all groups was not observed in the second sacrifice time. Obviously, the total rate of released IL-6 in second sacrifice time was declined in all groups. Results of IL-6 in the third sacrifice were shown that the levels of cytokines in the unstimulated lymphocyte cells of each group differed significantly (P-value: 0.000817) and the rest were insignificant.

In the first sacrifice time, PHA-stimulated lymphocytes secreted high-level of IFN-γ with a significant difference. Especially, IFN-γ secreted cytokine levels showed a significant difference between groups Pbs and Msc-Reo (P-value: 0.0461). The results of IFN-γ in the second sacrifice time revealed that PHA stimulated high IFN-γ secretion. In final sacrifice time, secreted IFN-γ levels increased significantly after PHA stimulation in Msc-Reo and Msc-Reo-nT groups compared to the other three Pbs, Reo, and Msc groups (P-value: 0.003).

Therefore, increasing exposure time in both oncolytic reovirus and MSCs in mice bodies could increase the rate of stimulation and secretion of IFN-γ cytokine level. Thus, undoubtedly several extra shots could lead to higher production and secretion of IFN-γ cytokine.

The secretion of IL-10 cytokine in control cells of Pbs and Msc groups showed significant differences with other groups, (P-value: 0.0002) and (P-value: 0.0005), respectively, in the first sacrificing.

The secretion of this cytokine was significantly different among stimulated lymphocytes by both PHA and E7 compared to control cells (not receiving antigens). It seems this difference was due to anti-inflammatory properties of the tumor and MSCs response.

In the second sacrifice time, only the level of IL-10 in the Msc group was significantly higher than other groups (P-value: 0.005). Both PHA and E7 stimulated higher secretion of IL-10.

In the last sacrifice time, no significant differences in secretion levels among groups were obtained. It seems that IL-10 is used by tumors for growth via a shift to TH2 in the absence of reovirus.

In the first sacrifice time, TGF-β in the control Pbs group was significantly different in comparison with the other groups (P-value: 0.018).

The secretion of TGF-β following PHA stimulation in the Pbs control group is significantly lower than the other two Reo and Msc groups (P-value: 0.046).

**Fig. 8:**
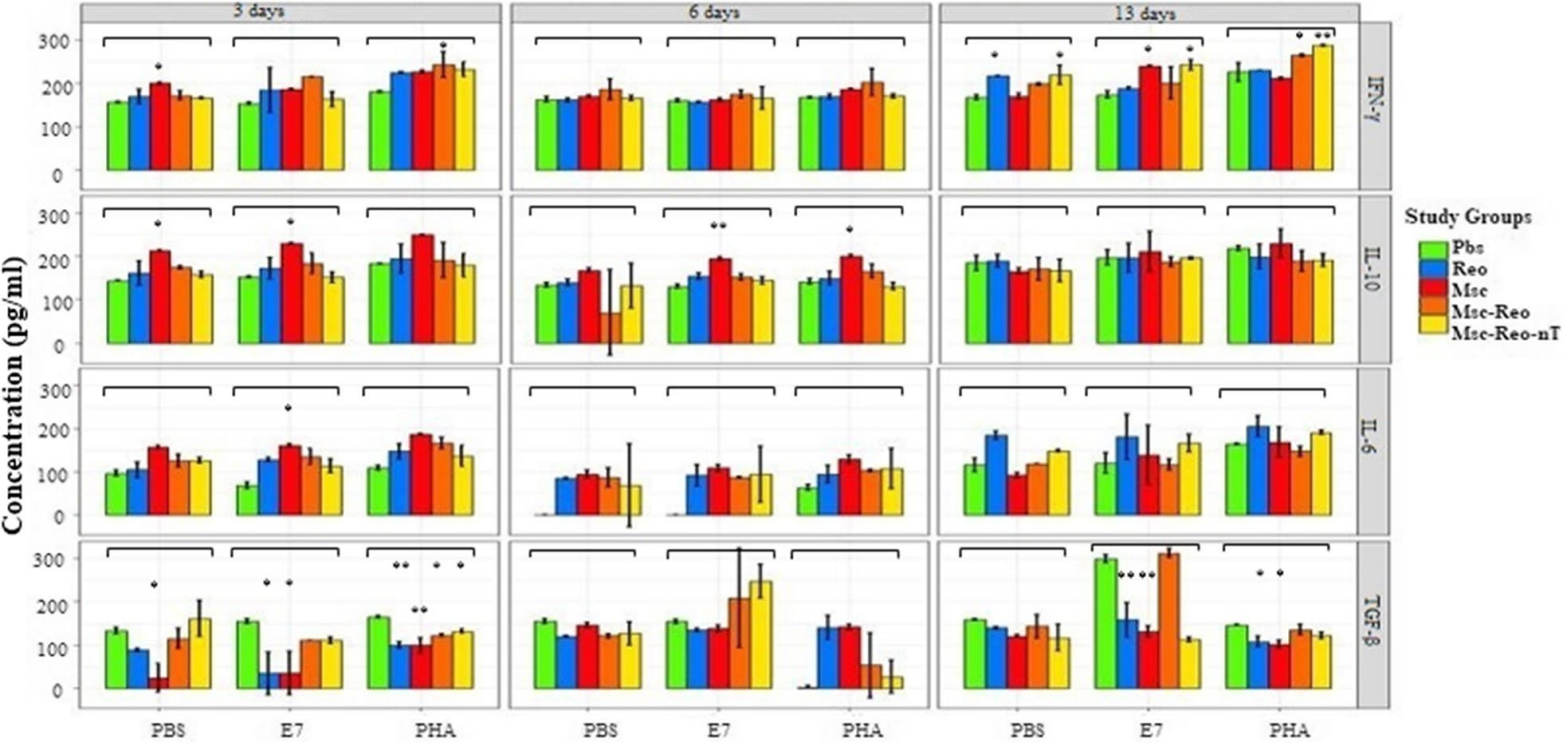
Determination of the splenic IL-6, IFN-γ, IL-10, and TGF-β cytokine secretion in response to stimulators. According to analyzed data, the robust response in the released cytokines level was observed after 3rd treatments. Reovirus had a direct effect on released IFN-γ level and an indirect effect on inflammatory cytokines when accompanying with MSCs its function was moderated. The experiment was performed in triplicate (*: P ≤ 0.05; **: P ≤ 0.01)

On the other hand, in the 3rd sacrifice time after stimulation with E7, the higher TGF-β level is secreted in Msc-Reo group compared to Reo, Msc, and Msc-Reo-nT groups (P-value: 0.00285, P-value: 0.00137, and P-value: 0.00085, respectively) (Fig. 8).

### Analysis of the distribution of Labeled-MSCs in vivo

**Fig. 9:**
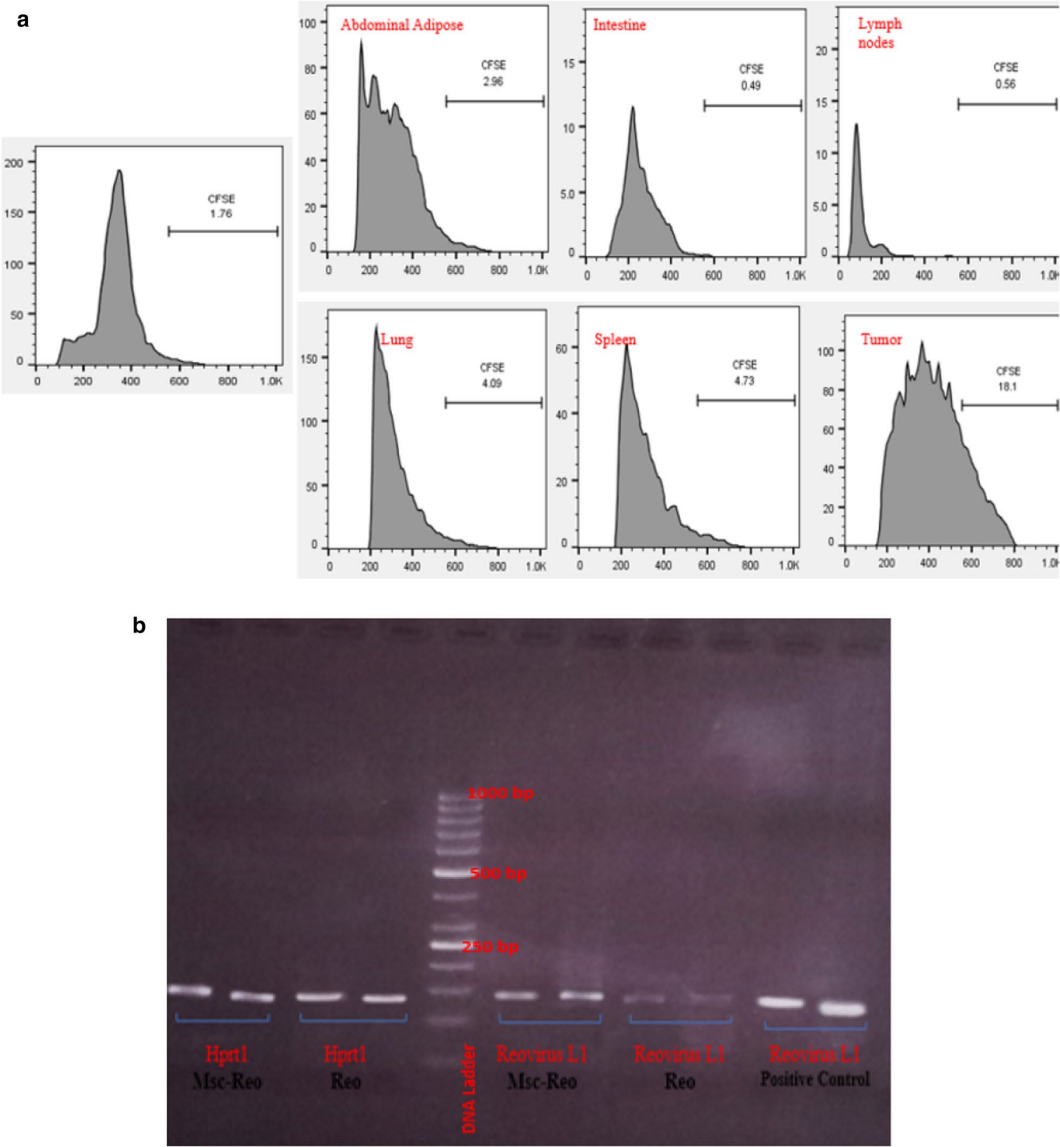
**a** The in vivo distribution of MSCs were traced in mice organs and **b** virus genome detection in tumors site. **a** After first accumulation in the lung, the I.V injected CFSE-Labelled MSCs were distributed in mice's body via blood circulating system and additionally in the tumor sites. Released soluble factors such as chemokines, cytokines, and growth factors from tumor cells could recruit MSCs to tumor sites (**b**). The results of PCR showed that the MSCs could act as a delivery vehicle to the tumor site. The size of the amplified L1 segment of reovirus was 135 bp length. To visualize the PCR product 2% agarose gel electrophoresis is used.

To evaluate the biodistribution of CFSE-labelled MSCs in mice body after I.V injection, several targeted organs of the animal were separated and performed based on the mentioned protocol in method and the results were assessed by flow cytometry. The presence of stained cells (labelled) in every organ was determined in comparison with the control group. The obtained information was concurrent with in vivo imaging information (unpublished data). The output of this method was implemented as follows: in the lung (4.09%), spleen (4.73%), tumor lateral lymph nodes (0.56%), intestine (0.49%), abdominal adipose (2.96%) and finally in the tumor site (18.1%) (Fig. 9a). Additionally, to verify the presence of a viral particle in tumor tissue, it was screened to find out the reovirus genome (L1 segment) via the PCR method (Fig. 9b). The reovirus genome was indicated in tumor tissue in both Msc-Reo and Reo groups.

The extracted viral genome from viral stock was used as a positive control and normalized by HPRT1 (138 bp length) as a housekeeper gene.

## Discussion

The current study was conducted based on the hypothesis that MSCs could be used as an ideal vehicle for oncolytic virus delivery to improve the outcome of cancer therapy. A fundamental requirement to use a cell as a virus delivery vehicle is that those cells must support the virus replication to produce infectious progeny [51]. Accordingly, based on previous research [50], AD-MSCs were susceptible and permissible to oncolytic reovirus infection, therefore, AD-MSCs assumed as a promising virus delivery vehicle.

The significant apoptosis induction that was observed in reovirus-infected MSCs (in vitro results) maybe due to the nuclear factor κB (NF-κB) activation by the virus [52–54] (Fig. 3). Following infection of MSCs with reovirus, the biology of cells tends to be altered.

MSCs have the great migration potential that could be altered as a result of infections. The important role of expressed CXCR4 in migration of MSCs toward bone marrow has been reported by Robert et al. [55, 56]. The CXCR4/MIF and CXCR4/CXCL12 axis had been reported as the key elements of MSC migration toward tumor cells [33, 57, 58]. By assessing the CXCR4, our data showed that reovirus has no devastating effect on migratory potency of infected-MSC, particularly in low MOI infection. NF-κB signaling pathway plays a critical role in this phenomenon. Therefore, as a result of the activation of the NF-κB signaling pathway via reovirus, the upward trend of CXCR4 was observed [59].

Tumor cells release various soluble chemokines and cytokines into the extracellular environment in interaction with other cells which can recruit MSCs and immune cells toward tumor sites [56] based on reports of previous studies, oncolytic reovirus can affect iron homeostasis (iron/ROS metabolism) in infected MSCs and consequently, it can cause increasing the CXCR4 expression on MSCs surface and enhance their migration potency [60–62].

Concurrently, to evaluate the ability of MSCs migration in vivo, we traced CFSE-labelled MSCs distribution into tumoral mice by flow cytometry 30 h post intravenous injection. Data revealed that MSCs were mainly accumulated in the lung, spleen, and tumor sites. After intravenous administration of MSCs via tail vein, initially, it can cause bulk-trapped MSCs in the lung and then their gentle redistribution to inflammation sites [63]. Several experiments used MSCs as a carrier to deliver oncolytic viruses into tumor cells had been reported with some successful results. Moreover, studies have revealed that MSCs' migration potency can be increased or decreased as a result of both viral and bacterial infections, depending on the nature of pathogens [19, 21, 59]. The enhancement of tumor-tropic migration potency in infected stem cells with the oncolytic virus was examined by Atique et al. They have reported that the significant difference was observed between infected neural stem cells and control groups in tumor-tropic migration potency [37].

In our study design, we should notice that the induced antiviral antibodies produced during treatments could impede the distribution of viruses into the tumor site. Therefore, to avoid this probable complication, we used AD-MSCs as a vehicle and all the treatment process was planned in a short period of time. Castleton et al. reported that oncolytic measles-loaded MSCs overcome preexisting neutralizing antiviral antibodies in both solid and hematological malignancies [64]. We encountered the induction of neutralizing antibodies through treatments that were proved by the poor outcomes in the Reo group.

The most important and effective mechanisms against both virus-infected cells and tumor cells are the cytotoxic activity of T lymphocytes and natural killer cells (NK). To evaluate the cytotoxicity response, LDH method was used in this study. The results of the LDH assay showed that the LDH level among groups in the first and second sacrifice time did not differ significantly. In the last sacrifice time, the highest rate of cytotoxicity was observed in Msc and Msc-Reo groups (P-value < 0.05) and surprisingly showed declined level in the Reo and Msc-Reo-nT groups (P-value > 0.05). It is presumed in Msc and Msc-Reo groups, the main circumstances to MSCs activity as the potent antigens presenting cells (APCs) is prepared via reovirus infection. MSCs have an inherent property of expressing MHC-I and subsequently can present viral and tumor antigens to CD8 + cytotoxic T lymphocytes (CTLs). Additionally, under an appropriate condition of stimulation, MSCs could act as specialized APC to present antigens to T-helper cells (TCD4 +). In a typical scenario, releasing of IFN-γ via NK-Cell, TH1 and CTL in response to viral infection may cause an appropriate tumoral antigens released by the destruction of infected-cancer cells. In this circumstance, the high concentration of IFN-γ can affect MSCs to alter as a potent APCs to present tumor antigens [33].

Atique et al. approved that MSCs can adversely impact on T-cells differentiation and function in co-cultural experimentations [65]. Theoretically, reovirus infection can influence on MSCs devastatingly due to the cell cycle arrest via protein σ1s and subsequently, the MHC-II expression on MSCs could not have a much promising sight in Msc-Reo group. In fact, viruses can recruit cellular machinery to accelerate the development of infection [66, 67].

The rates of apoptosis and autophagy among groups were assessed by flow cytometry and real-time PCR. The high levels of total apoptosis in tumoral tissues among different groups were observed in Msc and Msc-Reo groups. Previously, the overexpression of autophagy and apoptotic genes has been reported when the oncolytic virus applied [68, 69].

The current study revealed that the tumor size growing remained similar among groups during the initial monitoring, but 18 days after treatment the noticeable changes have occurred.

Subsequently in splenocytes that stimulated via specific antigen and mitogen, several cytokines were measured to find out a correlation between the efficacy of different treatments and subsequently cytokines release. The amount of IL-6 was not significantly different in approximately all treated groups compared to Pbs control group, except the Msc group which secreted the high-level IL-6. MSCs secrete a wide range of cytokines and chemokines in response to environmental stimulators [68]. When MSCs were infected by oncolytic reovirus, TNF-α [70, 71] and IL-6 [72, 73] released immensely in extracellular vesicles. These pro-inflammatory cytokines are involved in systemic inflammation and can promote both apoptosis and necrosis. However, some reports claimed that the secretion of IL-6 from MSCs in tumor sites can promote tumor growth by its regenerative activities [74]. High levels of released IFN-γ were recognized in both Msc-Reo and Msc-Reo-nT groups (ORV-loaded MSCs) and interestingly increased over time. The TH1 cells in these groups were strongly stimulated by ORV-loaded MSCs which interacted with tumor cells. IFN-γ released from TH1 acts as a main anti-tumoral defence cytokine and causes tumor suppression. Eventually, triggered signals by IFN-γ recruit immune mononuclear cells such as macrophages, which secrete inflammatory cytokines, to the tumor site. Zhang and colleagues reported that the high level of IFN-γ was released in treated mice with hUCMSCs-LV-IL-21 [75]. MSCs can promote tumor growth by dwindling the IFN-γ release from TH1 and increasing IL-4 expression from TH2. This phenomenon leads to the expansion of the regulatory T-cell population [33].

The high amount of anti-inflammatory IL-10 cytokines which related to MSCs modulatory role mainly detected in the Msc group, and can shift immune responses to regulatory T cells. The high level of TGF-β was observed in the Msc-Reo group especially when stimulated by E7 antigen. TGF-β could inhibit tumor progression via inducing cell arrest and triggering apoptosis [76].

Shah et al. reported that ICOVIR17-loaded MSCs showed inhibitory effects on tumor progress compared to direct usage of ICOVIR17 [10]. Castleton and colleagues showed that MSCs could be used as an effective carrier to deliver the oncolytic measles virus in the systemic route in the treatment of lymphoblastic leukemia [31]. Tanja et al. reported prolonged survival of mice with the lung tumor were observed when they received oncolytic adenovirus-loaded MSCs intravenously [51]. Inhibitory effect of MSCs on tumor progression has been reported by Khakoo et al. in which MSCs can downregulate the Akt signaling pathway in tumor cells [77]. The magnificent results in treating lung tumors and brain metastatic melanomas via HSV-loaded MSCs have been reported by Shah et al. [53].

Principally, the contradictory results in outcomes of MSCs usage are reported because of their dependence on MSCs origin, nature of tumor cells, tumor microenvironment, MSCs-secreted vesicles, the main route of administration, and so on. For instance, adipose-derived MSCs act in favor of the glioma cell line U87MG, whereas controversial results were described when the same cell line was treated with umbilical cord-derived MSCs [74, 78–82].

We did not observe any side effects due to I.V administration of MSC/Reovirus in mice, in all experiments. Some data previously reported that the administration of adenovirus intravenously may cause accumulation of viruses in the liver due to preexisting neutralizing antibodies [51]. In histopathology assessment of the present study, no pathological signs were observed in vital organs such as the liver.

In conclusion, the results of this study revealed that MSCs loaded with reovirus (MSC-RV) and MSCs can suppress tumor progression by several probable mechanisms that involved (i) NF-κB pathway (ii) Interferon-Stimulated Gene (ISG) expression (iii) apoptotic bodies formation (iv) direct viral particle effect (active or dead particles (v) as a transport vehicle (vi) down-regulation of WNT signaling pathway [21] (vii) down-regulating the expression of vascular endothelial growth factor (VEGF) [78, 82] (viii) activation and recruitment of CTL and TCD4 + and immune cells toward the tumor site.

All together also the Msc-Reo group had shown more anticancer effect compared to the Reo group, but the engagement of the immune system in the Msc group clearly was observed. This data shows the privilege of using the MSC-based vehicle to deliver the oncolytic virus to the site of the tumor, but the efficiency of the platform is not good enough and repeating the treatment process is essential.

Accordingly, our findings support the hypothesis that reovirus-loaded MSCs via both reducing tumor size and stimulated cellular immunity could be considered as cancer therapeutic cell-based approaches.

## Conclusion

Overall, based on these observations using mesenchymal stem cells platform as a cell carrier to deliver the oncolytic virus to tumor sites has relatively promising outputs and using this platform along with combination therapy may have more effective outcomes.

Finally, more related comprehensive research is required to clarify the engaging molecular interaction among MSCs, oncolytic viruses, and tumor cells in more detail.

## Data Availability

All data generated or analyzed during this study are included in the main body of this article.
